# Preprocessing Strategies for Animal Behavior Classification Using Inertial Sensors: Effects of Filtering, Normalization, and Data Representation

**DOI:** 10.3390/s26175353

**Published:** 2026-08-24

**Authors:** Magno do Nascimento Amorim, Késia Oliveira da Silva-Miranda

**Affiliations:** 1 Department of Biosystems Engineering, Escola Superior de Agricultura “Luiz de Queiroz”, Universidade de São Paulo (ESALQ/USP), Piracicaba 13418-900, São Paulo, Brazil; kosilva@usp.br; 2 Department of Agricultural Engineering, Instituto Federal Goiano (IFGoiano), Urutaí 75790-000, Goiás, Brazil

**Keywords:** accelerometer, animal behavior, machine learning, signal preprocessing, precision livestock farming

## Abstract

**Highlights:**

**What are the main findings?**

- Preprocessing effects in accelerometer-based animal behavior classification are not universal but depend on the interaction among data representation, learning algorithms, and signal characteristics.
- Simpler pipelines without filtering combined with Z-score standardization provided robust performance, while high-pass filtering reduced accuracy and increased computational cost.

**What are the implications of the main findings?**

- Filtering and normalization should be selected according to the machine learning algorithm rather than applied as mandatory steps in behavior-classification pipelines.
- Computationally efficient pipelines may provide promising candidates for future deployment of real-time precision livestock farming systems using embedded devices and Edge Computing approaches.

**Abstract:**

Automatic classification of livestock behaviors, including feeding, rumination, standing, lying, walking, and drinking, using wearable accelerometers has become an important tool in precision livestock farming (PLF). However, the influence of preprocessing strategies on classification performance and computational efficiency remains poorly understood. This study investigated the effects of filtering, normalization, data representation, and machine learning algorithms using five accelerometer datasets comprising 3,533,974 records collected from different livestock species and sampling frequencies. Four filtering strategies, three normalization methods, two data representations, and four machine learning algorithms were evaluated using a standardized pipeline. Model performance was assessed using weighted F1-scores together with statistical and computational analyses. The absence of filtering achieved the highest average performance, reaching a weighted F1-score of 0.773 with Random Forest, whereas high-pass filtering consistently reduced performance (minimum average F1 = 0.605 across sampling frequencies) while increasing computational cost. Z-score standardization improved the performance of scale-sensitive algorithms, increasing SVM performance by up to 8.6% compared with no normalization. Feature-based representations provided greater stability for conventional machine learning models, whereas raw signals generally benefited the 1D-CNN. These findings demonstrate that preprocessing strategies should be selected according to the learning algorithm, signal characteristics, and computational constraints rather than applied as universal procedures, providing methodological guidance for the development of efficient livestock behavior monitoring systems.

## 1. Introduction

Automatic animal behavior monitoring has become an important application of precision livestock farming (PLF), enabling the continuous monitoring of behaviors through wearable sensors combined with machine learning (ML) techniques. Among the different technologies available, triaxial accelerometers stand out due to their low cost, reduced energy consumption, high acquisition frequency, and ability to continuously record movement patterns across different species [1]. Consequently, a significant increase has been observed in studies using these sensors for the development of automatic monitoring systems, contributing to animal welfare, early disease detection, precision management, and decision support in animal production [2].

Despite these advances, transforming raw accelerometer signals into reliable behavioral information remains an important challenge. The signals recorded by sensors are influenced by several factors, including mechanical vibrations, electronic noise, device orientation, individual differences among animals, sensor placement, and environmental conditions. Therefore, different preprocessing steps are commonly applied before feature extraction and machine learning model training, aiming to reduce interference, standardize data, and improve the discrimination of behavioral patterns [3].

Behavior classification pipelines usually include different preprocessing steps, such as filtering, normalization, and data representation, followed by the training of classification algorithms. Although these steps can directly influence model performance, limited evidence is still available regarding their individual contributions and interactions [2]. A recent scoping review revealed that preprocessing choices vary considerably among studies and are frequently adopted without systematically evaluating their individual contributions or interactions. Consequently, substantially different preprocessing pipelines are employed across studies, making direct methodological comparisons difficult and limiting the development of evidence-based recommendations for accelerometer-based animal behavior classification [2].

Among preprocessing steps, signal filtering plays an important role. Low-pass and Butterworth filters are widely employed to reduce high-frequency noise, whereas high-pass filters are used to remove low-frequency components, particularly gravitational acceleration [4,5]. Simpler methods, such as Moving Average filters, are also used due to their ease of implementation and reduced computational cost [3], while more sophisticated approaches, such as Kalman filters and Wavelet-based techniques, have been applied in specific applications requiring greater preservation of temporal signal characteristics [6,7].

However, despite being widely adopted, filtering does not always provide consistent benefits for behavior classification. Riaboff et al. [8] observed a reduction in accuracy after applying high-pass filtering in dairy cattle, suggesting that removing static components may eliminate relevant information for discriminating behaviors. These findings indicate that different filtering strategies preserve distinct components of accelerometer signals, making their effects potentially dependent on species, monitored behavior, sampling frequency, and the algorithm used. Moreover, although filtering is routinely included in behavior-classification pipelines, few studies have quantitatively isolated its contribution to predictive performance, meaning that its benefits are often assumed rather than experimentally demonstrated [2].

Another widely employed preprocessing step is data normalization. Techniques such as Min-Max scaling and Z-score standardization are frequently applied to reduce scale differences among features and facilitate the training of machine learning models. Previous studies have reported improvements in algorithm stability, reduction in numerical issues, and greater consistency of results after applying these strategies [5,9,10,11]. Nevertheless, quantitative comparisons among normalization methods remain scarce, particularly across different machine learning algorithms. Consequently, direct comparisons between normalized and non-normalized pipelines are still limited, preventing practical recommendations regarding the most appropriate normalization strategy for different learning algorithms [2].

Besides filtering and normalization, the way sensor data are represented before classification can substantially influence model behavior. Conventional machine learning algorithms generally rely on handcrafted statistical features extracted from accelerometer signals, whereas deep learning architectures are capable of learning discriminative representations directly from raw signals. Although both approaches have demonstrated good performance in livestock applications, it remains unclear whether preprocessing strategies exert similar effects across these fundamentally different data representations [2,12].

Another aspect receiving increasing attention is the computational efficiency of behavior-classification pipelines. Recent advances in Edge Computing and Tiny Machine Learning (TinyML) have enabled machine learning models to be executed directly on embedded devices attached to animals, reducing dependence on cloud infrastructure while enabling continuous real-time monitoring. Under these conditions, preprocessing choices affect not only predictive performance but also processing time, inference latency, memory usage, and energy consumption, all of which directly influence the feasibility of deploying intelligent livestock-monitoring systems [13,14,15].

Although filtering, normalization, and data representation are routinely included in behavior-classification pipelines, they are generally evaluated independently or adopted without a systematic assessment of their combined effects. Consequently, it remains unclear which preprocessing strategies consistently improve classification performance, how these effects vary according to the learning algorithm and sampling frequency, and whether potential performance gains justify the additional computational cost required by more complex pipelines. Recent studies have further emphasized that preprocessing decisions can substantially influence both predictive performance and computational efficiency, and that their effects are highly dependent on the characteristics of the dataset and the learning algorithm employed [16]. Furthermore, the absence of standardized methodological guidelines hampers the reproducibility and comparability of machine learning studies, making it difficult to identify preprocessing strategies that can be consistently transferred across datasets and practical applications [17,18]. Addressing these questions is essential for establishing practical methodological guidelines that support the development of accurate, computationally efficient, and reproducible behavior-classification systems.

Accordingly, this study was designed to address the following research questions:

I. Does signal filtering improve animal behavior classification compared with using raw signals?
II. How do different normalization strategies affect the predictive performance of machine learning algorithms?
III. Do filtering and normalization strategies interact differently according to the classification algorithm and data representation?
IV. How does sampling frequency influence the effectiveness of preprocessing strategies?
V. Which preprocessing pipeline provides the best trade-off between predictive performance, statistical robustness, and computational efficiency?

Thus, this study aimed to systematically evaluate the effects of different filtering and normalization strategies on the predictive performance, statistical robustness, and computational efficiency of machine learning models applied to automatic animal behavior classification. Additionally, we investigated how these effects are influenced by the algorithm used, sampling frequency, and data representation, aiming to establish methodological guidelines and practical recommendations for developing behavior-classification pipelines using inertial sensors in PLF.

## 2. Materials and Methods

### 2.1. Dataset

Five accelerometer datasets obtained from different livestock species were used in this study. The datasets were selected because they represent diverse experimental conditions with respect to species, sensor placement, sampling frequency, behavioral repertoire, and sample size, allowing the evaluation of whether the effects of preprocessing strategies remain consistent across different animal monitoring scenarios. The datasets included sampling frequencies ranging from 1 to 25 Hz and comprised cattle, sheep, and goats monitored using wearable accelerometers positioned at different anatomical locations, including the jaw, limb, noseband, horn, and neck collar.

All datasets were organized into a standardized structure containing acceleration signals from the x, y, and z axes, along with their respective behavioral labels. When available, additional information, such as individual animal identification and experimental sequences, was preserved to enable validation strategies across individuals. This standardization was restricted to variable organization and nomenclature, without modifying the original signal values or behavioral labels provided by the dataset authors.

The original distributions of behavioral classes were preserved in all datasets, and no balancing techniques or artificial resampling methods were applied. This decision was intended to maintain the original characteristics of the datasets and evaluate model performance under conditions that more closely resemble real animal monitoring scenarios. The main characteristics of the datasets used in this study are presented in Table 1.

Due to the large data volume of Dataset 4, a stratified temporal-block subsampling strategy was applied before executing the classification pipelines. The original dataset contained 12,756,669 accelerometer observations, which were reduced to 1,020,896 observations after subsampling (approximately 8.0% of the original data volume). Subsampling was performed independently within each combination of animal identification, recording sequence, and behavioral class. Continuous temporal blocks of 60 s (1500 observations at 25 Hz) were generated, and blocks shorter than 5 s were discarded.

To preserve the temporal structure of the signals while maintaining biological diversity, candidate blocks were randomly shuffled (random seed = 42). The selection procedure initially prioritized blocks originating from different animals before additional blocks were included until the predefined sampling target was reached. A maximum of 250,000 observations was retained for each behavioral class, whereas classes containing fewer observations than this threshold were fully preserved. Consequently, all behavioral classes and animals were represented in the sampled dataset whenever possible.

This strategy was adopted because the objective of the present study was to compare the relative effects of preprocessing strategies on model performance rather than estimate the prevalence of behaviors in the original dataset. Therefore, subsampling reduced temporal redundancy [2], improved computational feasibility, and preserved the biological and behavioral diversity present in the original dataset. Only a single stratified subsample was generated because the objective was to obtain a computationally feasible dataset while maintaining representative temporal and biological variability, rather than to evaluate sampling variability across multiple independent subsamples.

When available, individual animal identification was used during model cross-validation. This strategy ensured that observations from the same individual were not simultaneously present in the training and testing sets, reducing the risk of information leakage and providing a more realistic estimate of model generalization to previously unseen animals [5].

### 2.2. Data Preprocessing Strategies

To evaluate the influence of preprocessing strategies on the performance of classification models, an experimental pipeline composed of different combinations of filtering and normalization methods applied to raw acceleration signals was developed. In addition to predictive performance, the impact of these strategies on the computational efficiency of the models was also evaluated. All pipelines were applied in a standardized manner across the evaluated datasets. All preprocessing and model development steps were implemented in Python 3.11.3, and details of the computational environment are provided in Section 2.7. After preprocessing, the signals were subjected to the same steps of segmentation, feature extraction, feature selection, model training, and statistical evaluation. This standardization allowed the observed differences to be attributed exclusively to the preprocessing strategies evaluated. In total, twelve preprocessing combinations were evaluated, resulting from the combination of four filtering strategies and three normalization strategies, allowing the assessment of both the isolated and combined effects of these techniques across different sampling frequencies, animal species, and sensor configurations. The filtering and normalization strategies are described in the following sections.

#### 2.2.1. Experimental Preprocessing Scenarios

The twelve experimental scenarios resulted from the combination of four filtering strategies and three normalization strategies (Table 2). The filtering strategies evaluated were: no filtering, Moving Average filter, Butterworth low-pass filter (LPF), and high-pass Butterworth filter. The normalization strategies included: no normalization, Min-Max normalization, and Z-score standardization.

Each combination defined a preprocessing pipeline applied to the signals before segmentation and the subsequent analysis steps. This factorial structure allowed the evaluation of both the individual effects of filtering and normalization and the interaction between these two steps. The scenario without filtering and without normalization was adopted as the baseline for comparison with the other evaluated pipelines.

#### 2.2.2. Signal Filtering

Three filtering strategies were evaluated, in addition to a scenario without filtering, as previously presented in Table 2. The first strategy consisted of applying a Moving Average filter, whose output was obtained using Equation (1).

(1) $$y\left[n\right]=\frac{1}{N}\;\sum_{i=0}^{N-1}x\;[n-i]$$

where *x*[*n − i*] represents the original signal value at sample *n − i*; *y*[*n*] corresponds to the filtered signal; and *N* represents the smoothing window size.

In this study, a three-sample window (N = 3) was used. This strategy performs local signal smoothing by averaging neighboring samples, and reducing small fluctuations and high-frequency components while preserving general movement patterns.

The second method employed a second-order low-pass Butterworth digital filter to attenuate high-frequency components and preserve the predominant patterns of animal movement. A normalized cutoff frequency of 0.30 relative to the Nyquist frequency was used.

The third method consisted of applying a second-order high-pass Butterworth filter, which was used to remove low-frequency components, mainly the gravitational component (static acceleration associated with gravity), emphasizing the dynamic components of body movement. For this strategy, a normalized cutoff frequency of 0.05 was used.

The Butterworth filters were implemented using the SciPy library (v1.17.1) through the scipy.signal.butter() and scipy.signal.filtfilt() functions. The use of the filtfilt() function allowed double-pass filtering (forward-backward filtering), eliminating temporal phase shifts commonly introduced by causal digital filters.

All filters were independently applied to the X, Y, and Z accelerometer axes. Each axis was processed individually before the subsequent steps of the classification pipeline. The filter order and normalized cutoff frequencies were kept constant across all datasets to ensure a consistent digital filtering procedure regardless of the native sampling frequency. Consequently, although the normalized filter characteristics remained the same, the corresponding cutoff frequencies in Hz and the effective temporal duration of the Moving Average filter differed according to the sampling frequency of each dataset. The equivalent temporal duration of the Moving Average filter and the corresponding Butterworth cutoff frequencies for each sampling frequency are presented in Table 3. Since the objective of this study was to evaluate preprocessing strategies as they are commonly implemented in accelerometer-based machine learning pipelines, rather than to compare physically equivalent filters across different sampling frequencies, the Moving Average filter was intentionally defined using a fixed number of samples, whereas the Butterworth filters were specified using normalized cutoff frequencies.

#### 2.2.3. Data Normalization

After the filtering step, three normalization conditions were evaluated, each independently applied to the X, Y, and Z accelerometer axes. In all experiments, the processing pipeline consisted of filtering, normalization, signal segmentation, data representation (raw signals or extracted features), and model training.

The first condition used the original signals without any scaling and served as the baseline for comparison. The second condition employed Min-Max normalization (Equation (2)), which rescales each variable to the interval between 0 and 1 while preserving the relative ordering of the observations [24]. The third condition employed Z-score standardization (Equation (3)), centering each variable around zero and scaling it to unit variance. This transformation is commonly adopted to reduce differences in variable scale and improve the performance of algorithms sensitive to feature magnitude [25].

(2) $$x^{\prime }=\frac{x-x_{min}}{x_{max}-x_{min}}$$

(3) $$z=\frac{x-\mu }{\sigma }$$

where $x^{\prime }$ is the normalized value, x is the original variable value, $x_{min}$ and $x_{max}$ are the minimum and maximum values of the variable, respectively, z is the standardized value, μ is the mean of the variable, and σ is the standard deviation.

Normalization parameters were calculated independently for each accelerometer axis using only the training partition of each cross-validation iteration. The resulting parameters were subsequently applied to the corresponding test partition, preventing information leakage while preserving the original distribution of unseen data.

#### 2.2.4. Data Segmentation

Accelerometer time series were segmented into fixed-duration windows of 5 s with 50% overlap. This window size was selected based on previous studies reporting better behavioral separability using short- to medium-duration windows, typically ranging from 5 to 10 s [2]. The use of overlapping windows helps reduce information loss at window boundaries and improves the representation of behavioral transitions [26].

For datasets with low sampling frequencies, where the exact definition of 50% overlap was not possible due to the discretization of the number of samples per window, the window shift was rounded to the integer number of samples closest to 50% of the window size. Incomplete windows at the end of the time series were discarded, ensuring consistency in the sample size used for model training.

In the scenario using raw signals, each window was represented by the original acceleration values from the three axes (X, Y, and Z) over time. Therefore, the number of input variables per sample directly depended on the sampling frequency of each dataset. Considering a 5 s window, the number of variables was defined as the number of samples per axis multiplied by the three accelerometer axes. For example, in datasets sampled at 1 Hz, each window contained 5 samples per axis (totaling 15 variables), whereas in datasets sampled at 10 Hz, each window contained 50 samples per axis (150 variables). For higher frequencies, such as 25 Hz, each window could contain up to 125 samples per axis, resulting in 375 variables in the raw data scenario.

The label assigned to each window corresponded to the predominant behavioral class among the samples contained within the respective segment, determined by the modal value of the labels present in the window. This strategy reduces the influence of short-duration behavioral transitions and ensures the association of a single behavior with each segment used for model training.

#### 2.2.5. Feature Extraction

Feature extraction represents a fundamental step in classical accelerometer-based behavior-classification pipelines, allowing time series to be represented by a reduced set of attributes capable of summarizing relevant movement patterns, reducing data dimensionality, and facilitating the training of machine learning algorithms [2,12,27].

In this study, features from the time and frequency domains were extracted, as well as movement intensity metrics derived from the combination of the three accelerometer axes. Initially, widely used statistical features were calculated separately for each axis (X, Y, and Z), including mean, standard deviation, minimum value, maximum value, range, variance, median, Root Mean Square (RMS), skewness, and kurtosis.

In addition to features calculated individually for each axis, the Signal Vector Magnitude (VM) was estimated. The same statistical features described above were calculated from the vector magnitude, allowing the overall activity level of the animal to be represented independently of sensor orientation.

To characterize movement intensity, Signal Magnitude Area (SMA), Overall Dynamic Body Acceleration (ODBA), and Vectorial Dynamic Body Acceleration (VeDBA) were also calculated. These metrics are widely used to quantify locomotor activity and energy expenditure in animal behavior studies [2,28].

Frequency-domain features were obtained by applying the Fast Fourier Transform (FFT) to the acceleration vector magnitude. The spectral representation allowed the extraction of spectral energy, spectral entropy, and dominant frequency, which are descriptors capable of representing periodic signal components frequently associated with rhythmic and repetitive behaviors.

In total, 46 features were extracted for each segmented window, including statistical descriptors from individual axes, vector magnitude features, movement intensity metrics, and spectral attributes. This combination was selected because it integrates features widely used in behavior-classification studies based on inertial sensors, covering temporal, biomechanical, and spectral information [2,12,27]. The complete list of extracted features, as well as the mathematical formulation of the accelerometer-derived metrics, is provided in Supplementary Material Table S1.

#### 2.2.6. Feature Selection

After feature extraction, a feature selection step was performed to reduce data dimensionality, remove redundant information, and decrease the computational cost of the pipeline while maintaining the predictive performance of the models [29,30].

Feature selection was based on variable importance estimated by Random Forest models. For each cross-validation iteration, a Random Forest model was fitted using only the training data. The importance of each feature was estimated based on the Mean Decrease in Impurity (MDI), which quantifies the contribution of each variable to reducing impurity in the decision tree nodes during forest construction [30,31]. Although MDI is computationally efficient and widely adopted, it may introduce bias in the estimated importance of variables with different statistical characteristics.

Features were then ranked according to their importance values, and the 12 most relevant variables were selected to compose the reduced feature subset. This subset corresponds to approximately 25% of the 46 extracted features and was adopted to obtain a compact representation of the original feature space while substantially reducing dimensionality and computational cost.

To avoid data leakage, the entire feature selection procedure was performed independently within each cross-validation fold. Therefore, feature importance estimation was obtained exclusively from the training data and subsequently applied to the corresponding test set.

### 2.3. Machine Learning Models

Four machine learning algorithms were evaluated: Random Forest (RF) [31], Support Vector Machine (SVM) [32], k-Nearest Neighbors (KNN) [33], and a One-Dimensional Convolutional Neural Network (1D-CNN) [34]. These models were selected because they represent different supervised learning paradigms, including decision tree-based methods, separation margin-based methods, distance-based methods, and deep learning approaches, while also being widely employed for behavior classification using inertial sensors [2].

The same hyperparameters were maintained throughout all experiments to ensure that performance differences were primarily associated with the evaluated preprocessing strategies rather than classifier optimization. The selected configurations correspond to commonly adopted default or near-default settings available in the Scikit-learn and TensorFlow/Keras implementations. Dataset-specific hyperparameter tuning was intentionally not performed because the objective of this study was to compare preprocessing strategies under a standardized experimental protocol.

The Random Forest model was configured with 200 decision trees, the Gini splitting criterion, random_state = 42, and parallel processing enabled (n_jobs = −1). The Support Vector Machine model was implemented using a Radial Basis Function (RBF) kernel, regularization parameter C = 1.0, gamma = scale, and random_state = 42. The k-Nearest Neighbors model was configured with k = 5 neighbors.

The 1D-CNN architecture consisted of two one-dimensional convolutional layers containing 32 and 64 filters, respectively, both using the Rectified Linear Unit (ReLU) activation function, a kernel size of 3, and the same padding. Subsequently, a MaxPooling1D layer with a window size of 2, a GlobalAveragePooling1D layer, a fully connected layer with 64 neurons and ReLU activation, a Dropout layer with a rate of 0.20, and a Softmax output layer containing the number of neurons corresponding to the number of behavioral classes in each dataset were applied.

The input representation of the 1D-CNN depended on the adopted data representation. For compatibility with the classical machine learning algorithms, raw temporal windows were temporarily stored as one-dimensional vectors and subsequently reshaped to their original (window length × 3) structure before being supplied to the 1D-CNN. For the raw-signal representation, each input sample consisted of a five-second temporal window containing synchronized acceleration signals from the x-, y-, and z-axes, which were provided to the network as three independent input channels. Consequently, the input tensor dimensions were (5 × 3), (50 × 3), and (125 × 3), corresponding to the five-second windows extracted from datasets sampled at 1, 10, and 25 Hz, respectively.

For the feature-based representations, the extracted variables were reshaped into one-dimensional vectors before being supplied to the network, resulting in input dimensions of (46 × 1) for the complete feature set and (12 × 1) for the selected feature subset. In this case, the convolution operation no longer modeled temporal dependencies in the original accelerometer signals but instead learned local relationships among neighboring features according to the adopted feature ordering. The same network architecture was used for all data representations to ensure a consistent comparison among preprocessing strategies.

The 1D-CNN was trained using the Adam optimizer [35], categorical cross-entropy loss function, batch size of 32, and a maximum limit of 20 epochs. In each fold, 20% of the training data were randomly reserved for internal neural network validation and used exclusively to monitor training and define the Early Stopping criterion [36]. Training stopped when the validation loss (val_loss) did not decrease for three consecutive epochs, and the weights corresponding to the lowest validation loss were automatically restored.

### 2.4. Model Evaluation

Model performance was evaluated using five-fold cross-validation, following the same partitioning strategies adopted in the previous chapter. Data segmentation into overlapping windows was performed prior to model training, and the validation strategy depended on the metadata available for each dataset. For Datasets 1 and 2, which did not contain individual animal identifiers or recording-session metadata, Stratified K-Fold cross-validation was applied, preserving the proportion of behavioral classes in each partition and following the validation protocols adopted in the original studies from which these datasets were obtained. For Datasets 3–5, which included individual animal identification, the GroupKFold strategy was used, ensuring that all windows generated from the same animal remained within the same fold, thereby preventing records from the same individual from being simultaneously assigned to the training and testing sets. This approach reduces the risk of performance overestimation caused by dependency among observations from the same individual and provides a more realistic estimate of model generalization capacity for animals not observed during training [5].

Model performance was quantified using accuracy, precision, recall, and weighted F1-score metrics (Equation (4)). Accuracy represents the overall proportion of correct classifications, whereas precision and recall evaluate, respectively, the reliability of positive predictions and the ability of the model to correctly identify each behavior. The weighted F1-score was adopted as the primary metric for model comparison because it combines precision and recall while accounting for the frequency of each behavioral class, making it particularly suitable for multiclass classification problems with imbalanced class distributions. To verify the robustness of the results, Macro F1-score and balanced accuracy were also computed for all experimental scenarios. As these complementary metrics showed trends consistent with those obtained using the weighted F1-score (Supplementary Material Table S2), the weighted F1-score was retained as the primary metric for the analyses presented in the main text.

(4) $${F1}_{weighted}=\sum_{i=1}^{C}\frac{n_{i}\;}{N}\times {F1}_{i}$$

where C is the total number of classes, $n_{i}$ is the number of samples belonging to class i, N is the total number of samples, and $F1_{i}$ is the F1-score computed for class i.

All metrics were calculated independently for each cross-validation fold. Statistical analyses and comparisons among preprocessing strategies were performed using the weighted F1-score, while the additional metrics were used to verify the consistency of the observed performance patterns across the evaluated pipelines.

### 2.5. Computational Efficiency

In addition to predictive performance, the computational efficiency of the different preprocessing pipelines was evaluated considering metrics related to processing cost and feasibility for application in real-time animal monitoring systems. Preprocessing time, model training time, inference time, and the number of input variables used in each experimental scenario were evaluated.

Preprocessing time corresponded to the interval required to execute the filtering and normalization steps before signal segmentation and data representation. Training time was defined as the period required for complete model fitting using the training set corresponding to each cross-validation fold.

Inference time was calculated as the average time required to classify one sample after model training and was expressed in milliseconds per sample (ms sample^−1^). All execution times were measured using the time.perf_counter() function from the Python standard library (Python version 3.11.3), which is designed for high-resolution measurements of execution time.

In addition to temporal metrics, the number of input variables used in each experimental scenario was recorded. This metric was employed as an indirect indicator of pipeline computational complexity, since strategies based on raw signals and extracted features result in different input dimensions for machine learning algorithms, directly influencing training and inference costs.

The combined evaluation of these metrics allowed the comparison of different pipelines not only in terms of predictive performance but also regarding the balance between accuracy and computational efficiency. This approach provides relevant information for selecting preprocessing strategies aimed at applications involving embedded devices, Edge Computing architectures, TinyML, and precision livestock farming systems operating under processing, memory, energy consumption, and latency constraints.

### 2.6. Statistical Analysis

The statistical analysis was conducted separately to evaluate the effects of filtering and normalization strategies on the performance of classification models. Since the comparisons involved multiple preprocessing strategies evaluated on the same sets of results obtained in each cross-validation fold, statistical tests for repeated measures were applied. Because the objective of this study was to compare the relative effects of preprocessing strategies rather than optimize the recognition of individual behavioral classes, the weighted F1-score was adopted as the primary performance metric. This metric was selected because it provides an overall assessment of classification performance while preserving the original class distributions of the evaluated datasets, which were intentionally maintained to represent realistic livestock-monitoring scenarios. Statistical analyses were performed independently for each Dataset × Model × Data Representation combination, using the weighted F1-score values obtained from the five cross-validation folds as paired repeated measurements for comparing the evaluated preprocessing strategies. This approach allowed each experimental configuration to be analyzed independently, avoiding comparisons across heterogeneous datasets, learning algorithms, or data representations.

Initially, global differences among filtering strategies were evaluated using the non-parametric Friedman test, considering the weighted F1-score values obtained in each cross-validation fold as paired units of comparison. Cross-validation fold results were used as repeated measurements because each fold provides a consistent estimate of model performance under the same preprocessing strategy, enabling paired comparisons among preprocessing methods within each experimental configuration. This test was selected because it is appropriate for comparing multiple treatments under repeated measures without assuming data normality.

When significant differences were identified (*p* < 0.05), multiple pairwise comparisons among strategies were performed using the Wilcoxon signed-rank test with Bonferroni correction to control the increased probability of Type I error resulting from multiple comparisons. The same procedure was adopted to evaluate the effects of different normalization strategies. The independent analysis of filtering and normalization steps allowed the specific contribution of each preprocessing component to the performance of behavior-classification models to be quantified.

In addition to significance tests, Kendall’s coefficient of concordance (Kendall’s W) was calculated for each Friedman test to quantify the effect magnitude of the evaluated strategies. Therefore, result interpretation considered both statistical significance and effect size, allowing statistically significant differences to be distinguished from differences with greater practical relevance. Although cross-validation folds are not fully independent observations because they originate from repeated partitions of the same dataset, they were treated as paired repeated measurements to enable consistent comparative analyses among preprocessing strategies within each experimental configuration. This limitation is acknowledged in the Discussion. All statistical tests were conducted adopting a significance level of 5% (α = 0.05).

### 2.7. Computational Environment

All analyses were conducted using the Python programming language (version 3.11.3). Data processing, preprocessing pipeline implementation, feature extraction and selection, model training, and statistical analyses were performed using established scientific libraries from the Python ecosystem.

The main libraries used included Pandas (v2.2.2) and NumPy (v1.26.4) for data manipulation and processing; SciPy (v1.17.1) for digital filter implementation, frequency-domain analysis, and statistical tests [37]; and Scikit-learn (v1.8.0) for data normalization, cross-validation, feature selection, and implementation of Random Forest, Support Vector Machine, and k-Nearest Neighbors algorithms, as well as performance metric calculation [38].

The One-Dimensional Convolutional Neural Network (1D-CNN) architecture was implemented using TensorFlow (v2.21.0) and Keras (v3.13.2). Result visualization was performed using the Matplotlib library (v3.10.8), while computational progress monitoring was conducted using tqdm (v4.66.5). All experiments were executed in a local environment using the Visual Studio Code (version 1.131.0) development environment on a computer equipped with the Windows 11 operating system, an Intel x64 processor, and 8 GB of RAM. No dedicated Graphics Processing Unit (GPU) was used, and all steps were executed exclusively on the CPU.

## 3. Results

### 3.1. Effect of Filtering Strategies

**Figure 1:**
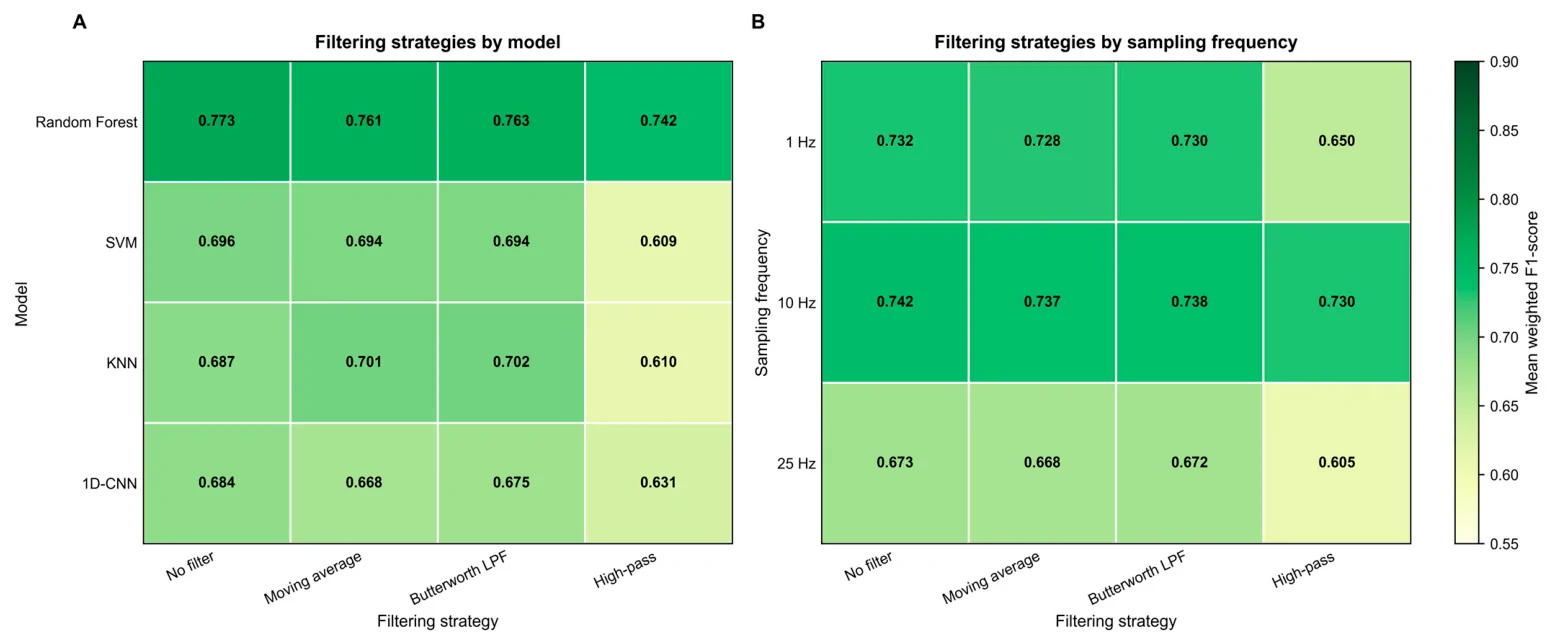
(**A**) Effect of filtering strategies on the average performance of Random Forest (RF), Support Vector Machine (SVM), k-Nearest Neighbors (KNN), and 1D-Convolutional Neural Network (1D-CNN) models. (**B**) Effect of filtering strategies considering datasets collected at 1, 10, and 25 Hz.

Supplementary Material Table S3 presents the complete results obtained for all combinations among datasets, machine learning models, filtering strategies, and normalization methods evaluated. Overall, the effect of filtering varied according to the dataset and algorithm used. However, a consistent trend toward higher performance was observed for pipelines without filter application, whereas the high-pass filter was associated with the lowest F1-score values (Figure 1).

Among the evaluated algorithms (Figure 1A), Random Forest achieved the highest average F1-score values regardless of the filtering strategy. The highest average performance was observed for Random Forest without filter application (F1-score = 0.773), whereas the lowest performance was obtained for SVM combined with the high-pass filter (F1-score = 0.609). Overall, differences among no filtering, Moving Average, and Butterworth filters were small, while the high-pass filter resulted in performance reductions for most algorithms.

When results were grouped according to sampling frequency (Figure 1B), a similar trend was observed. The absence of filtering achieved the highest mean weighted F1-score across the five cross-validation folds across the three evaluated frequencies. Datasets collected at 10 Hz showed the highest overall performances (F1-score = 0.742 without filtering), whereas the lowest average value was observed for data collected at 25 Hz using the high-pass filter (F1-score = 0.605).

The complete statistical results are provided in Supplementary Material Table S4, while Table 4 summarizes the frequency of scenarios with significant differences among filtering strategies and their respective effect sizes. Considering all evaluated combinations, 120 Friedman tests and 522 multiple comparisons using the Wilcoxon test were performed.

The statistical analysis showed that the influence of filtering strategies varied according to the characteristics of each dataset. Because the statistical analyses were performed independently for each dataset, the reported results describe the effectiveness of the filtering strategies within each classification scenario rather than direct comparisons among datasets. For Datasets 1 and 2, significant differences among filtering strategies were observed in almost all evaluated scenarios, with predominantly strong effect sizes (Kendall’s W = 0.69–0.93). In contrast, filtering had a more limited influence on Dataset 3, where only 29.2% of the evaluated scenarios showed significant differences and effect sizes were generally smaller, particularly for Random Forest (W = 0.26) and 1D-CNN (W = 0.23). Datasets 4 and 5 exhibited an intermediate response, with filtering strategies producing moderate to strong effects depending on the evaluated model.

Despite the global differences identified by the Friedman test, pairwise comparisons using the Wilcoxon signed-rank test with Bonferroni correction did not identify significant differences between specific filters (Supplementary Material Table S4).

Overall, these results indicate that filtering influences the performance of behavior classification; however, its effects depend on dataset characteristics and the algorithm employed. In general, additional filtering strategies did not result in consistent performance improvements, whereas the high-pass filter showed the strongest tendency to reduce the F1-score.

### 3.2. Effect of Normalization Strategies

**Figure 2:**
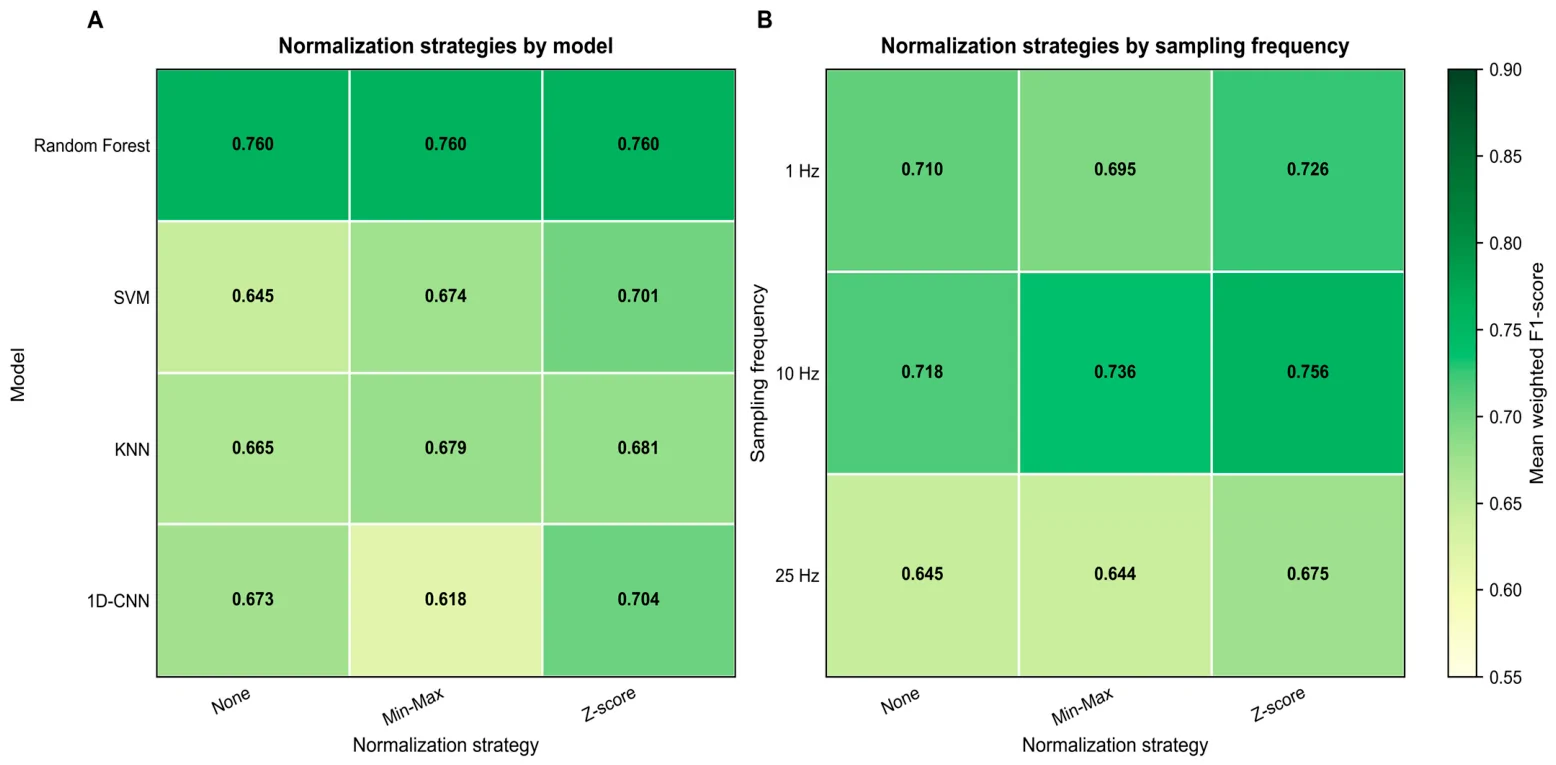
(**A**) Effect of normalization strategies on the average performance of Random Forest (RF), Support Vector Machine (SVM), k-Nearest Neighbors (KNN), and 1D-Convolutional Neural Network (1D-CNN) models. (**B**) Effect of normalization strategies considering datasets collected at 1, 10, and 25 Hz.

Figure 2A,B present the effects of normalization strategies considering the different machine learning models and sampling frequencies evaluated, respectively. Overall, the impact of normalization was dependent on the algorithm used, with greater influence observed for models sensitive to feature scaling. Among the evaluated strategies, Z-score standardization showed the most consistent performance, achieving superior or equivalent results compared with the other approaches in most evaluated scenarios.

The analysis by algorithm (Figure 2A) demonstrated different responses to normalization strategies. Random Forest showed nearly constant performance regardless of the transformation applied, maintaining average F1-score values close to 0.760. In contrast, SVM showed greater sensitivity to normalization, with average increases of approximately 4.5% using Min-Max normalization and 8.6% using Z-score standardization compared with the scenario without normalization.

KNN also showed improvement after normalization, although with a smaller magnitude. For the 1D-CNN, Z-score standardization increased the average performance by approximately 4.6%, whereas Min-Max normalization reduced the F1-score by approximately 8.2%.

When results were grouped according to sampling frequency (Figure 2B), Z-score standardization achieved the highest average F1-score values across all evaluated frequencies. Compared with the scenario without normalization, this strategy provided average increases of approximately 2.3%, 5.3%, and 4.7% in datasets collected at 1, 10, and 25 Hz, respectively. Min-Max normalization showed less consistent behavior, with lower average performances in datasets collected at 1 and 25 Hz.

The complete statistical results are provided in Supplementary Material Table S4, while Table 5 summarizes the frequency of scenarios with significant differences among normalization strategies and their respective effect sizes.

The statistical analysis confirmed that the influence of normalization was strongly associated with the algorithm used. Random Forest showed significant differences in only two of the 40 evaluated scenarios, with small effect sizes (Kendall’s W = 0.14–0.27), indicating low sensitivity to feature scaling. In contrast, SVM showed significant differences in 32 of the 40 scenarios, with predominantly strong effect sizes (W = 0.64–0.87). The 1D-CNN also showed a strong influence of normalization, with significant differences in 24 of the 40 scenarios and effect sizes ranging from 0.53 to 0.70. For KNN, the effects were intermediate, ranging from moderate to strong (W = 0.33–0.61).

Although the Friedman test identified significant global differences in several scenarios, pairwise comparisons using the Wilcoxon signed-rank test with Bonferroni correction did not indicate significant differences between specific pairs of normalization strategies (Supplementary Material Table S4).

Overall, these results demonstrate that the effect of normalization mainly depends on the characteristics of the machine learning algorithm. While tree-based models, such as Random Forest, showed low dependence on feature scale, distance-based, optimization-based, and deep learning algorithms were more influenced by normalization, with Z-score standardization showing the most robust behavior across the evaluated scenarios.

### 3.3. Performance of Combinations Between Filtering and Normalization Strategies

**Figure 3:**
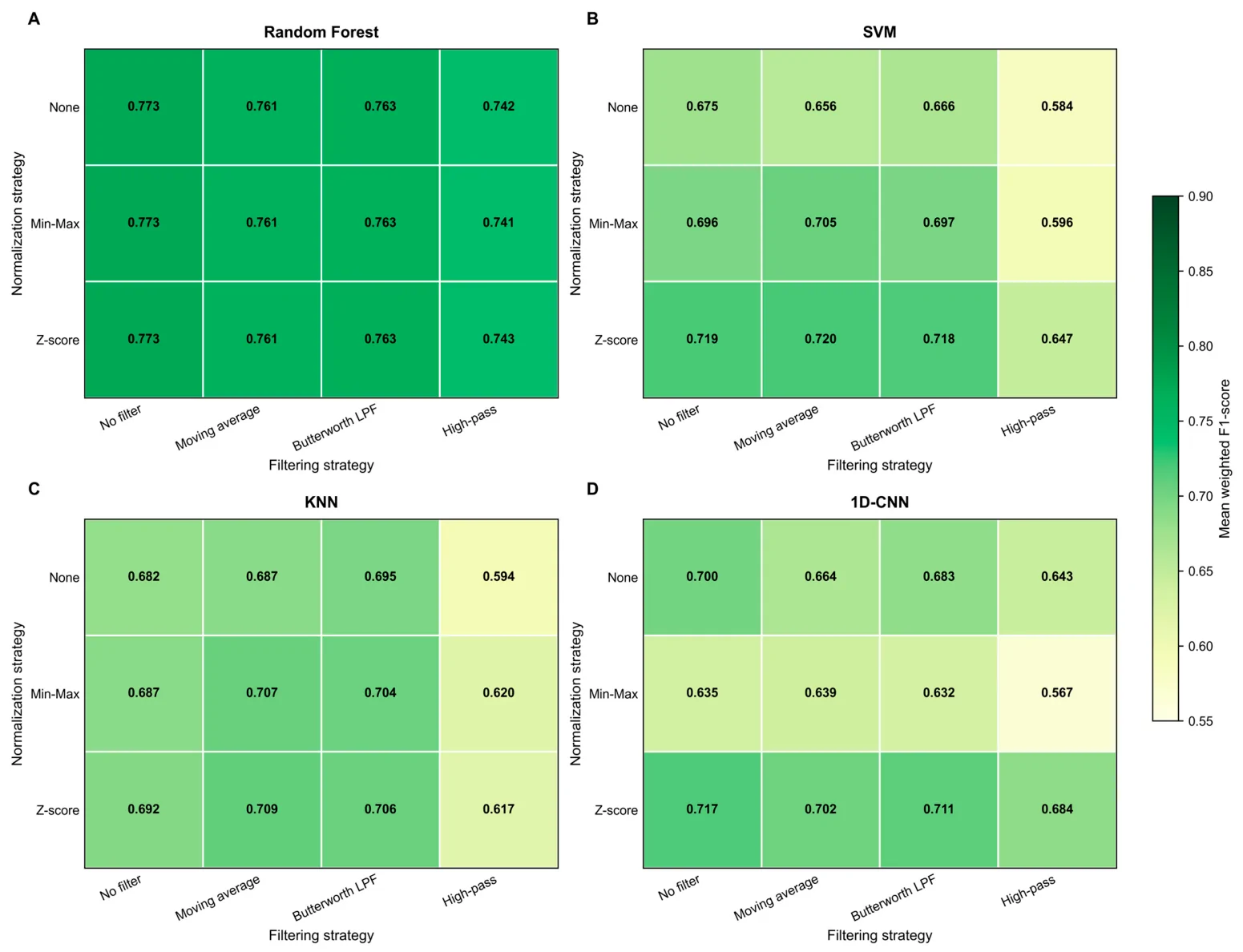
Interaction between filtering and normalization strategies on the average performance of the models: (**A**) Random Forest (RF); (**B**) Support Vector Machine (SVM); (**C**) k-Nearest Neighbors (KNN); and (**D**) One-Dimensional Convolutional Neural Network (1D-CNN).

Figure 3 presents the average performance obtained for all combinations between filtering and normalization strategies considering the four evaluated models. Panels A, B, C, and D correspond to the Random Forest (RF), Support Vector Machine (SVM), k-Nearest Neighbors (KNN), and One-Dimensional Convolutional Neural Network (1D-CNN) models, respectively. Overall, the impact of preprocessing strategies was dependent on the algorithm used, with greater influence observed for models sensitive to feature scaling.

The combined analysis of the strategies demonstrated that no single preprocessing combination was superior for all evaluated algorithms. However, some consistent patterns were observed. Z-score standardization was associated with the highest-performing combinations in most models, whereas the high-pass filter resulted in the lowest average F1-score values regardless of the normalization strategy applied.

Random Forest (Figure 3A) showed high stability across the different preprocessing combinations, maintaining similar performance regardless of the normalization strategy applied. The highest average F1-score was observed in scenarios without filter application (F1-score = 0.773), whereas combinations involving the high-pass filter showed the lowest performances.

In contrast, SVM showed greater sensitivity to the evaluated strategies (Figure 3B). The highest performances were consistently achieved with Z-score normalization, regardless of whether no filtering (F1-score = 0.719), Moving Average (F1-score = 0.720), or Butterworth low-pass filtering (F1-score = 0.718) was applied. Conversely, the lowest performance was observed for the High-pass filter without normalization (F1-score = 0.584), followed by the combination of High-pass filtering and Min-Max normalization (F1-score = 0.596). These results indicate that Z-score normalization substantially improved model performance, whereas High-pass filtering consistently degraded classification accuracy, particularly in the absence of an effective normalization strategy.

For KNN (Figure 3C), combinations involving smooth filtering strategies associated with normalization showed similar performances. The highest values were obtained using Moving Average + Z-score (F1-score = 0.709), Moving Average + Min-Max (0.707), and Butterworth + Z-score (0.706), indicating a lower influence of the filtering strategy when combined with appropriate normalization methods.

For the 1D-CNN (Figure 3D), the best performances were obtained using No filter + Z-score (F1-score = 0.717) and Butterworth + Z-score (0.711). In contrast, the combination High-pass + Min-Max presented the lowest performance among all evaluated scenarios (F1-score = 0.567).

Table 6 summarizes the combinations associated with the highest and lowest average performances for each algorithm, as well as the percentage difference between these configurations.

The sensitivity to preprocessing strategies varied substantially among algorithms. Random Forest showed high robustness, with only a 4.3% difference between the best and worst evaluated configurations. In contrast, SVM, KNN, and 1D-CNN showed differences of 23.3%, 19.4%, and 26.5%, respectively, demonstrating greater dependence on preprocessing steps. Overall, these results indicate that the choice of filtering and normalization strategies should consider the algorithm used. Although pipelines based on the absence of filtering combined with Z-score standardization provided the best overall balance among the evaluated models, the magnitude of the benefits varied according to the characteristics of each algorithm.

### 3.4. Interaction Between Data Representation and Preprocessing Strategies

**Figure 4:**
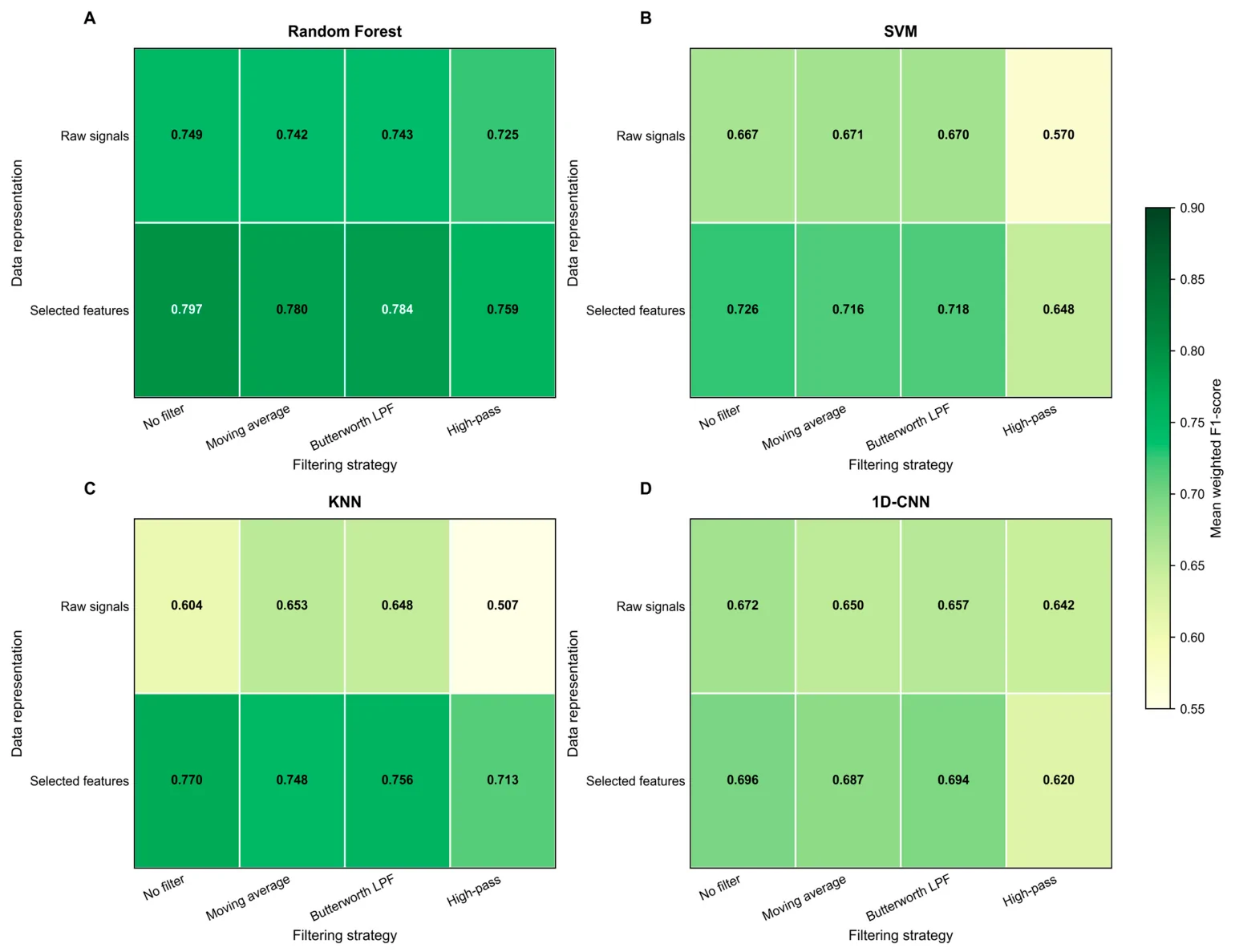
Effect of data representation on the performance of filtering strategies for the models: (**A**) Random Forest; (**B**) Support Vector Machine; (**C**) k-Nearest Neighbors; and (**D**) One-Dimensional Convolutional Neural Network. White and black numbers are used only to improve text contrast against darker and lighter background colors, respectively, and do not represent additional information.

**Figure 5:**
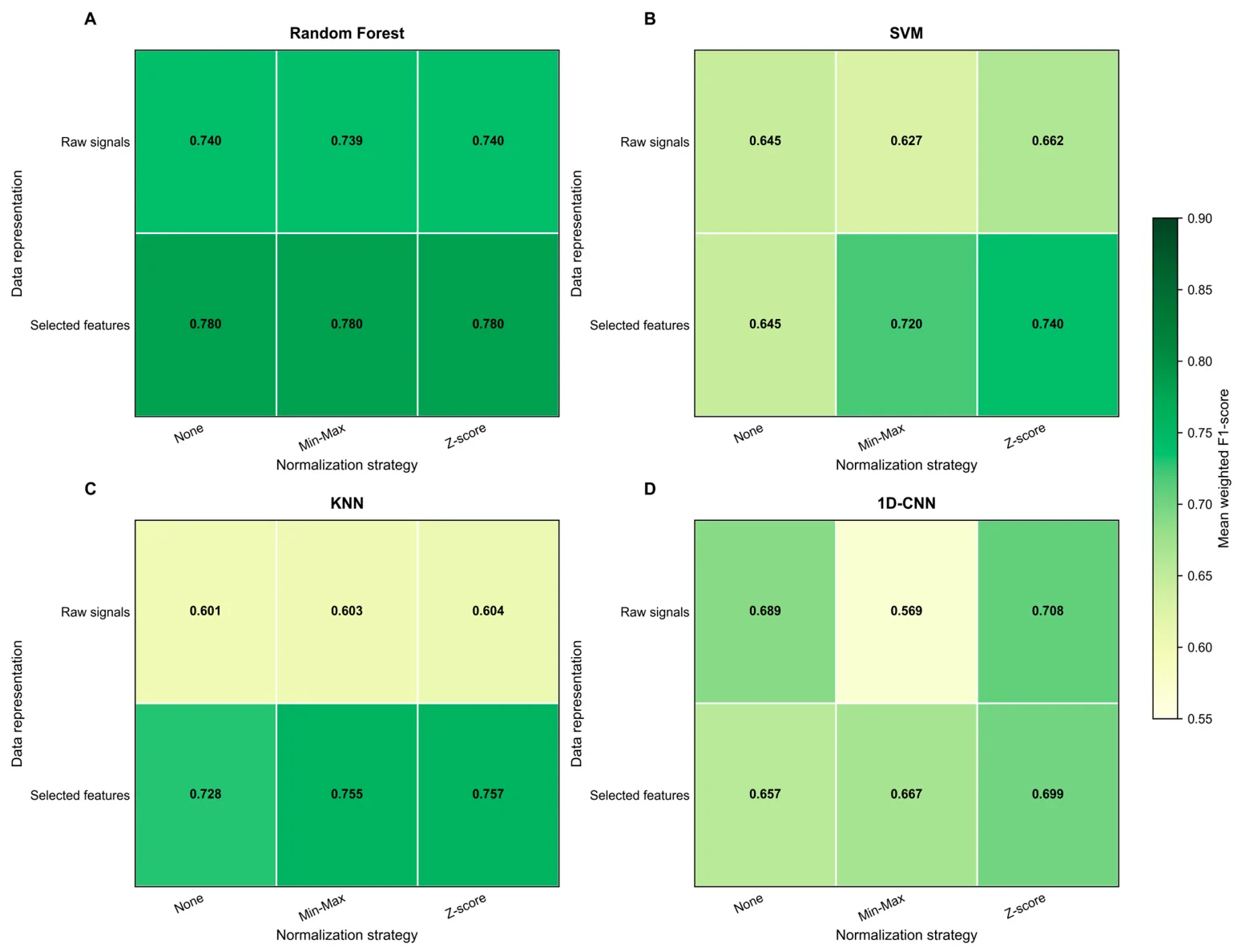
Effect of data representation on the performance of normalization strategies for the models: (**A**) Random Forest; (**B**) Support Vector Machine; (**C**) k-Nearest Neighbors; and (**D**) One-Dimensional Convolutional Neural Network.

Data representation modified the effect of preprocessing strategies, indicating that the choice between raw signals and extracted features influences the response of algorithms to filtering and normalization steps. Overall, the use of selected features provided greater performance stability for conventional machine learning algorithms, whereas the 1D-CNN showed a distinct behavior due to its ability to learn representations directly from temporal signals. Figure 4 and Figure 5 present these results.

Figure 4 demonstrates that the influence of filtering strategies was modified by data representation. For Random Forest, SVM, and KNN models, the use of selected features resulted in the highest average F1-score values and reduced the differences among filtering strategies, indicating greater stability of these models against variations in the preprocessing pipeline.

The best combinations for these algorithms were observed using selected features without filter application, with average F1-score values ranging from 0.696 to 0.797. In addition, differences among no filtering, Moving Average, and Butterworth filters were reduced after feature extraction and selection, indicating that feature-based representation made the models less dependent on previous filtering steps.

In contrast, the 1D-CNN exhibited a different response pattern from the conventional algorithms. Under most filtering conditions, selected features achieved slightly higher average F1-score values than raw signals, whereas raw signals showed a relative advantage only when combined with high-pass filtering. These results suggest that the influence of filtering on convolutional networks depends on the interaction between the filtering strategy and the adopted data representation.

Figure 5 demonstrates that the influence of normalization was also dependent on data representation. For Random Forest and KNN models, the use of selected features achieved higher performance than raw signals regardless of the normalization strategy applied.

For SVM, Z-score standardization promoted the highest performances in both evaluated representations, reinforcing the strong influence of feature scaling on this algorithm. Conversely, Min-Max normalization showed lower consistency, especially when applied directly to raw signals.

The 1D-CNN again showed a different response compared with the other algorithms. In scenarios without normalization and using Z-score standardization, direct processing of raw signals achieved higher performance than selected features, indicating that preserving the original temporal structure favored the automatic learning of representations by the convolutional network.

Overall, these results demonstrate that the choice of data representation modifies the influence of preprocessing strategies. While conventional algorithms consistently benefited from selected features, the response of the 1D-CNN depended on the interaction between data representation and the adopted preprocessing strategy. In particular, raw signals produced better performance under some normalization strategies, whereas selected features remained competitive under most filtering conditions.

### 3.5. Computational Efficiency

**Figure 6:**
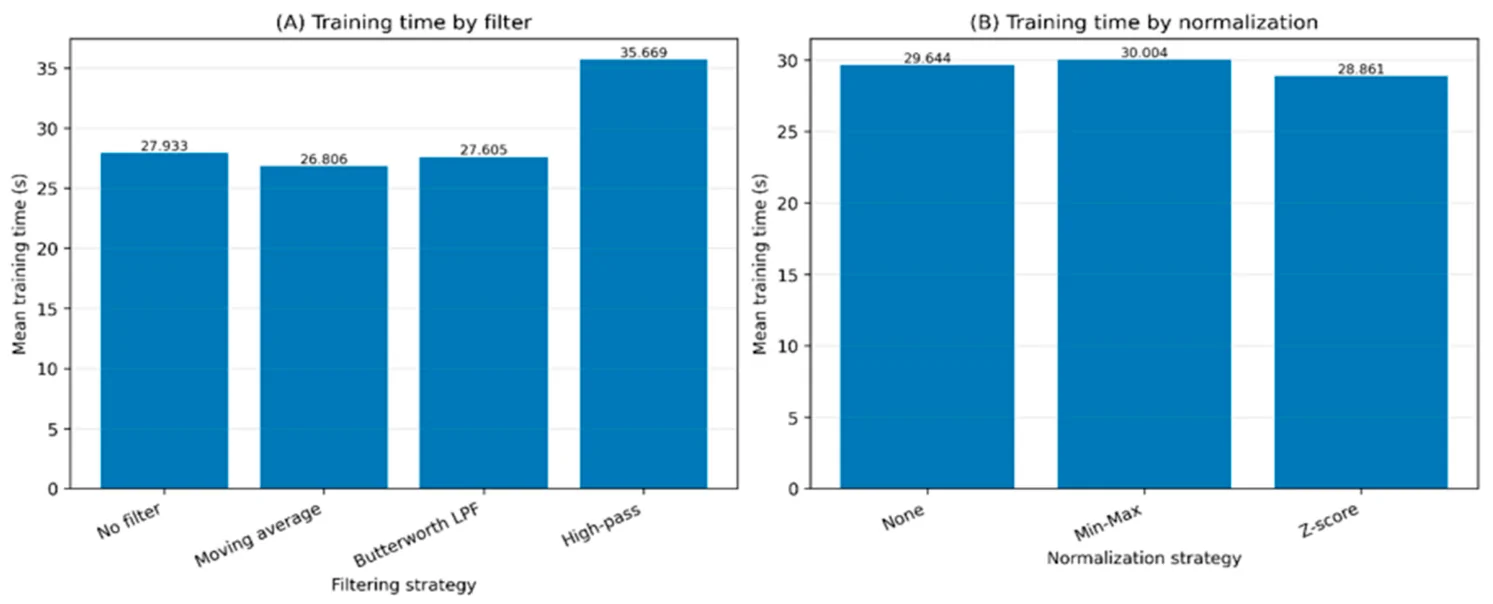
Average training time considering (**A**) filtering strategies and (**B**) normalization methods.

**Figure 7:**
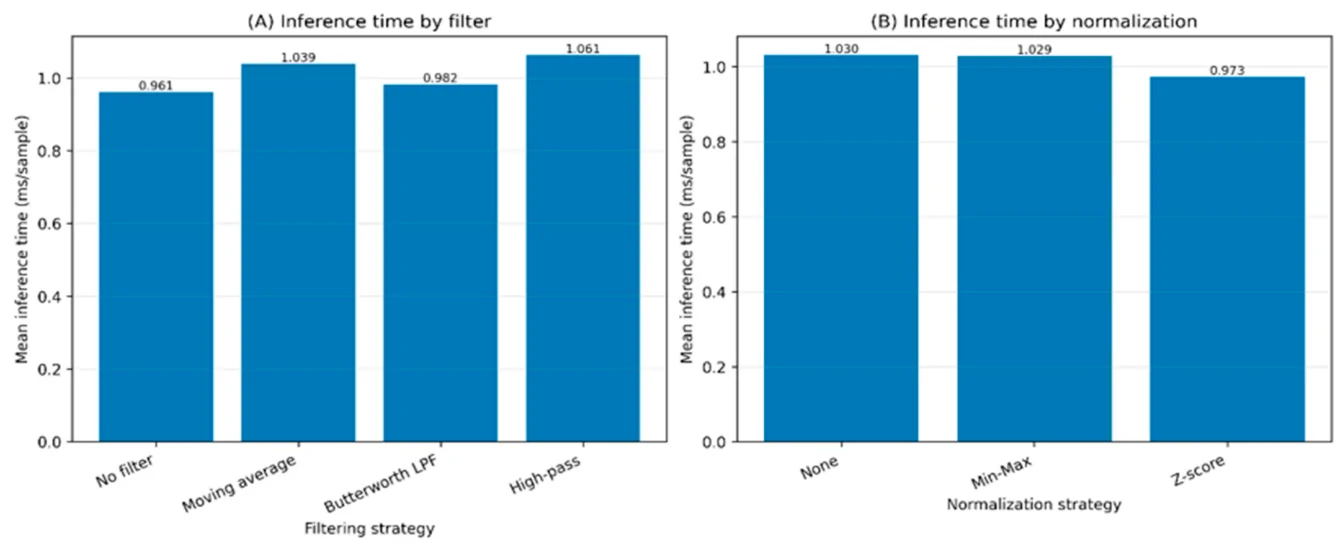
Average inference time considering (**A**) filtering strategies and (**B**) normalization methods.

**Figure 8:**
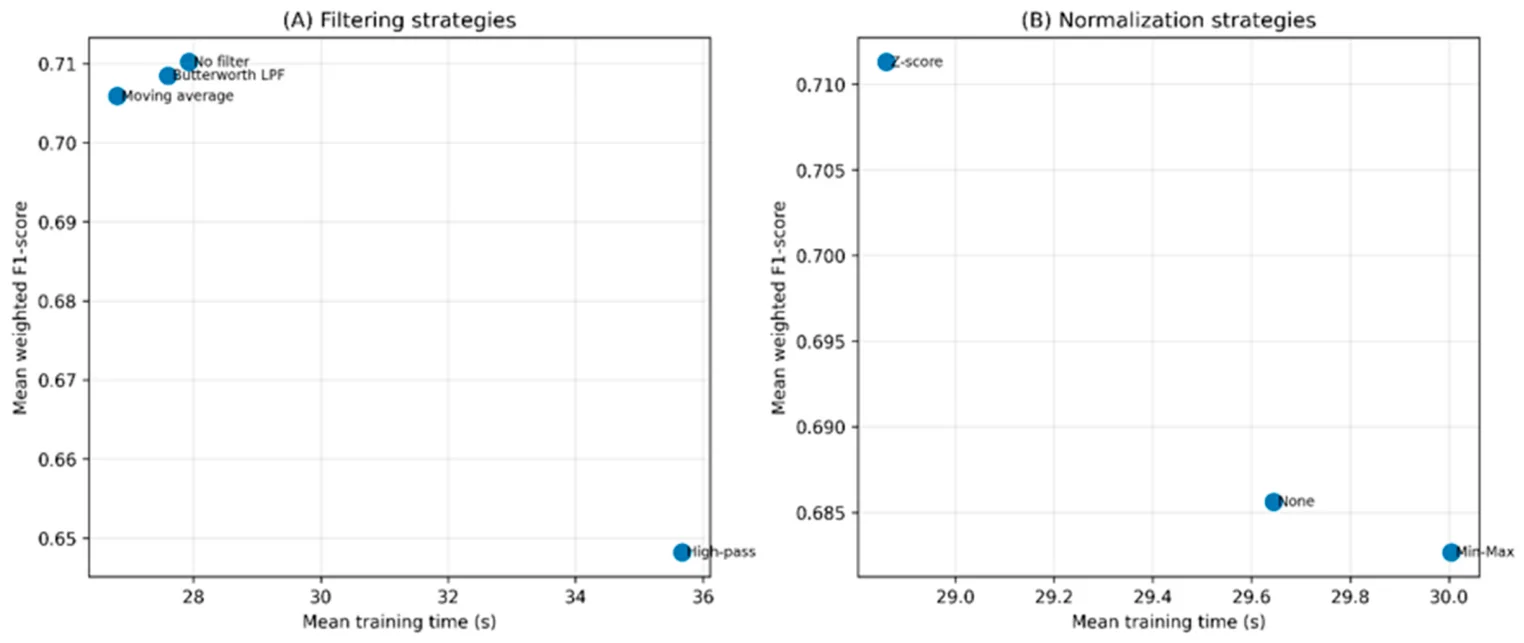
Relationship between predictive performance (F1-score) and average training time considering (**A**) filtering strategies and (**B**) normalization methods.

The complete computational efficiency results, considering all combinations among datasets, filtering strategies, normalization methods, data representations, and machine learning models, are provided in Supplementary Material Table S5. As a summary of these results, Figure 6 and Figure 7 present the average training and inference times, respectively, while Figure 8 demonstrates the relationship between predictive performance and computational cost.

Computational efficiency was mainly influenced by the filtering strategies applied (Figure 6A). The high-pass filter showed the highest average training time (35.669 s), whereas the Moving Average filter presented the lowest time (26.806 s). The scenarios without filtering and using Butterworth showed intermediate values and were similar to each other. The effects of normalization on training time were less pronounced (Figure 6B). Z-score standardization presented the lowest average time (28.861 s), whereas Min-Max normalization showed the highest computational cost (30.004 s).

For inference time, the absolute differences among strategies were smaller (Figure 7). Among the evaluated filters, the absence of filtering showed the lowest average inference time (0.961 ms per sample), whereas the high-pass filter presented the highest value (1.061 ms per sample). Regarding normalization, Z-score standardization again showed the lowest average inference time (0.973 ms per sample), whereas no normalization and Min-Max normalization presented similar values (1.030 and 1.029 ms per sample, respectively).

The combined analysis between performance and computational cost demonstrated important differences among the evaluated strategies (Figure 8). The high-pass filter simultaneously presented lower predictive performance and higher average training time, characterizing it as the least efficient strategy among the evaluated filters. Conversely, no filtering, Moving Average, and Butterworth presented similar F1-score values but with lower computational costs.

Among normalization methods, Z-score standardization showed the best balance between performance and efficiency, combining higher average F1-score values and lower training time. Min-Max normalization presented lower relative efficiency, whereas the absence of normalization showed intermediate behavior.

The statistical analysis confirmed that filtering had a greater influence on training time than normalization. Significant differences among filtering strategies were observed in 65 of the 120 evaluated scenarios (54.2%), with a strong average effect size (Kendall’s W = 0.53). In comparison, normalization produced significant differences in 49 of the 160 scenarios (30.6%), with a moderate average effect size (W = 0.43). For inference time, the influence of the evaluated strategies was less pronounced. Significant differences were detected in 54 of the 120 filtering scenarios and 53 of the 160 normalization scenarios, both associated with moderate effect sizes, indicating no clear predominance of either preprocessing strategy. To facilitate the comparison between filtering and normalization regarding computational efficiency, Table 7 summarizes the statistical results obtained for training and inference times.

Considering predictive performance and computational efficiency together, the results demonstrate that more complex preprocessing pipelines did not necessarily result in better overall performance. Instead, the absence of filtering or the use of simple filtering strategies combined with Z-score standardization represented the most efficient alternatives among the evaluated scenarios, providing a favorable balance between predictive accuracy and computational cost. These characteristics make such pipelines particularly attractive for embedded systems and real-time livestock-monitoring applications.

## 4. Discussion

### 4.1. Simpler Preprocessing Pipelines Can Outperform More Complex Approaches

Accelerometer signal filtering and data normalization are frequently incorporated into behavior-classification pipelines to reduce noise, minimize scale differences among features, and improve the training of machine learning models [2]. However, the present findings indicate that adding preprocessing steps does not necessarily improve predictive performance. Instead, the effectiveness of filtering and normalization depends on the characteristics of the signal, the learning algorithm, and the intended application.

Across most evaluated scenarios, omitting signal filtering produced equivalent or superior performance compared with the remaining strategies, whereas the high-pass filter consistently yielded the lowest F1-score values. Differences among no filtering, Moving Average, and Butterworth filtering were generally small, suggesting that increasingly complex filtering approaches provide limited benefits when raw accelerometer signals already preserve sufficient behavioral information.

Previous studies support these observations. Riaboff et al. [8] observed a reduction in performance after applying high-pass filtering in cattle, suggesting that removing static acceleration may eliminate relevant information associated with posture and low-frequency movements. Likewise, Mauny et al. [39] found that certain filtering strategies reduced the ability of models to classify goat behaviors, reinforcing that removing signal components does not always lead to performance improvements. Together, these findings indicate that filtering should be considered a signal-dependent step rather than a mandatory procedure in classification pipelines based on inertial sensors.

A possible explanation for this behavior is that components removed during filtering may contain important discriminative information for specific behaviors. Although high-pass filters can reduce components associated with gravity and slow signal variations, this information may also represent postural changes and movement patterns relevant for classification. This interpretation is consistent with studies demonstrating that low-frequency components of inertial signals contribute to activity characterization and that their preservation may improve the recognition of behavioral patterns [40,41].

Beyond predictive performance, reducing preprocessing steps also improved computational efficiency. The absence of filtering reduced processing costs without compromising behavior classification, which is an important characteristic for applications involving embedded devices and continuous monitoring systems. This result aligns with the increasing need to develop efficient models for PLF applications based on local processing and devices with energy and computational constraints [14].

Normalization exhibited a different pattern from filtering. Among the evaluated methods, Z-score standardization produced the most consistent results, particularly for algorithms sensitive to feature scaling. This contrast reinforces that filtering and normalization influence different stages of the pipeline: whereas filtering modifies the temporal structure of the signal, normalization affects the numerical distribution of the extracted features.

The benefits of Z-score standardization have been reported in studies involving sensor-based behavior classification and are associated with reducing scale differences among variables and improving stability during model training [5,42]. In contrast, although Min-Max normalization is frequently used, its dependence on extreme values can make it more sensitive to variability among different datasets and sensors [2,9]. This characteristic may explain the less consistent behavior observed for this strategy in the present study.

The interpretation of the filtering results should also consider that the preprocessing parameters were standardized in terms of digital implementation rather than equivalent physical duration. Specifically, the Moving Average filter was defined using a fixed three-sample window, whereas the Butterworth filters were implemented using normalized cutoff frequencies relative to the Nyquist frequency. Consequently, the effective temporal duration of the Moving Average filter and the corresponding cutoff frequencies in Hz varied according to the sampling frequency of each dataset. Therefore, the observed results should be interpreted as reflecting the performance of commonly adopted digital filtering strategies rather than physically equivalent temporal filters. Future studies should investigate whether biologically equivalent filtering configurations produce similar trends across datasets acquired at different sampling frequencies.

Taken together, these findings indicate that increasing preprocessing complexity does not necessarily improve automatic behavior classification using inertial sensors. Instead of supporting a universally optimal pipeline, the results demonstrate that preprocessing decisions should reflect the characteristics of the learning algorithm, the accelerometer signals, and the computational constraints of the target application. Within the scenarios evaluated here, pipelines combining no filtering (or simple filters) with Z-score standardization provided the most favorable balance between predictive performance, robustness, and computational efficiency, making them attractive candidates for real-time livestock-monitoring systems.

### 4.2. The Influence of Preprocessing Depends on the Machine Learning Algorithm

One of the main findings of this study was that preprocessing strategies affected the evaluated algorithms differently. Random Forest maintained nearly identical performance regardless of the normalization strategy, whereas SVM, KNN, and 1D-CNN consistently benefited from Z-score standardization. These findings indicate that preprocessing effectiveness is largely determined by the learning mechanism of each algorithm rather than by the dataset alone.

This interpretation agrees with previous studies reporting that the benefits of normalization are algorithm-dependent because different learning methods exhibit distinct sensitivities to feature scaling and data distribution [43,44]. Similar conclusions have also been reported in machine learning applications beyond livestock monitoring, reinforcing that preprocessing should be selected according to the operating principles of the algorithm rather than adopted as a universal procedure.

The stability observed for Random Forest can be explained by the structure of the algorithm. Decision trees partition the feature space using splitting criteria, such as the Gini index, rather than distance metrics or gradient-based optimization [31]. Consequently, linear transformations such as Min-Max normalization and Z-score standardization preserve tree structure and have little influence on predictive performance.

In contrast, SVM was considerably more sensitive to feature scaling because differences in variable magnitude directly affect kernel calculations and the resulting decision boundaries. Standardizing the features reduces this imbalance, facilitating optimization and improving model generalization [45].

KNN follows a similar principle because classification is entirely based on distances between samples. Without normalization, variables with larger numerical ranges dominate distance calculations and reduce the contribution of other informative features. Z-score standardization mitigates this effect, explaining the performance gains observed in the present study [43,46].

Although 1D-CNN does not explicitly rely on distance metrics, its training depends on iterative optimization of neural network weights. Large differences in input distributions may hinder convergence, whereas standardized inputs promote more stable optimization. This interpretation is consistent with normalization strategies commonly adopted in deep learning to improve training stability [47].

Taken together, these findings demonstrate that the importance of preprocessing increases with the sensitivity of the learning algorithm to feature scaling and statistical distributions. Tree-based models remained robust across preprocessing strategies, whereas algorithms relying on distance metrics, kernel functions, or iterative optimization benefited more consistently from normalization. Rather than recommending a single preprocessing strategy, these results support selecting preprocessing according to the learning mechanism of each algorithm while balancing predictive performance, robustness, and computational efficiency.

### 4.3. Data Representation Modulates the Effectiveness of Preprocessing Strategies

Data representation markedly influenced the effectiveness of preprocessing strategies. Whereas selected features increased the robustness of traditional machine learning algorithms to different filtering and normalization approaches, the response of the 1D-CNN depended on the interaction between data representation and the adopted preprocessing strategy. Raw signals tended to provide superior performance under specific normalization settings, whereas selected features remained competitive under most filtering conditions. These findings indicate that preprocessing effectiveness depends not only on the learning algorithm but also on how behavioral information is represented before classification.

Feature extraction is one of the most widely adopted strategies in inertial-sensor applications because it transforms raw time series into a compact set of attributes describing relevant signal patterns [12]. Besides reducing dimensionality, feature extraction can remove redundancies and provide representations that are better suited to conventional machine learning algorithms. This trend is also evident in animal behavior classification, where feature-based approaches remain predominant [2].

The present results reinforce this trend. For Random Forest, SVM, and KNN, selected features reduced the influence of filtering and normalization, producing more stable performance across preprocessing pipelines. This suggests that feature extraction concentrates much of the discriminative information contained in the original signals, reducing the dependence on transformations applied to raw data.

Unlike conventional algorithms, 1D-CNN automatically learns hierarchical representations directly from raw signals, reducing the need for manually engineered features [48]. Consequently, preserving the original temporal structure may allow the network to exploit temporal dependencies that are partially attenuated after feature extraction. In the present study, however, this advantage depended on the adopted preprocessing strategy rather than occurring consistently across all evaluated scenarios. Similar behavior has been reported in wearable-sensor applications, where deep learning architectures often outperform conventional approaches when operating directly on raw accelerometer signals [49,50]. Likewise, Sameh et al. [51] showed that deep learning architectures generally exploit raw-signal representations more effectively, whereas conventional algorithms depend more strongly on the quality of engineered features. Together with the present results, these studies support the interpretation that raw signals and extracted features favor distinct learning paradigms rather than representing universally superior alternatives.

Taken together, these findings highlight data representation as a key component of behavior-classification pipelines using inertial sensors. Rather than selecting preprocessing strategies independently, pipeline design should jointly consider the interaction among data representation, preprocessing strategy, and the learning mechanism of the classification algorithm, as the most suitable representation may vary according to the adopted preprocessing pipeline.

### 4.4. Influence of Sampling Frequency on the Effects of Preprocessing

Sampling frequency also influenced the response of machine learning models to preprocessing strategies. Although the evaluated datasets differed simultaneously in animal species, sensor placement, and monitored behaviors, the results indicate that temporal resolution should be considered when designing behavior-classification pipelines.

Previous studies have shown that the effect of sampling frequency depends on the temporal dynamics of the monitored behaviors, with higher frequencies generally favoring rapid movements and lower frequencies being sufficient for more stable activities [52,53]. Consistent with this interpretation, filtering exerted a stronger influence on datasets collected at 1 Hz, where the reduced temporal resolution makes the loss of signal information caused by smoothing or component removal proportionally more relevant for behavioral discrimination.

Higher acquisition frequencies also capture a greater amount of high-frequency components and potentially redundant information. Consequently, the effectiveness of filtering depends on the relationship between the monitored behavior and the frequency content of the recorded movements. Unlike filtering, which directly modifies the temporal and spectral composition of accelerometer signals [41,54], normalization primarily affects the numerical distribution of input variables. Consequently, normalization showed less dependence on sampling frequency and remained consistently beneficial for algorithms sensitive to feature scaling, particularly SVM, KNN, and 1D-CNN [44,55].

These findings have practical implications for the development of real-time animal monitoring systems. Although higher sampling frequencies can provide more detailed representations of movement, they also increase the volume of processed data, energy consumption, storage requirements, and data transmission demands, which are critical factors for battery-powered wearable devices [53,56].

Taken together, these findings indicate that sampling frequency should be selected according to the monitored behavior, the learning algorithm, and the computational constraints of the target application rather than following a universal recommendation. Balancing temporal resolution with predictive performance, energy consumption, storage requirements, and processing capacity is particularly important for wearable sensors, Edge Computing, and continuous livestock-monitoring systems.

### 4.5. Potential Implications for Edge Computing and Real-Time Herd Monitoring

Automatic animal behavior classification has evolved from approaches based exclusively on offline processing toward embedded systems designed to support continuous monitoring under field conditions. In this context, predictive performance should be considered together with computational cost, latency, memory requirements, energy consumption, and operational autonomy when evaluating the potential practical adoption of these technologies in precision livestock farming systems.

Edge Computing and TinyML have emerged as promising approaches for executing machine learning models closer to the data-acquisition source, potentially reducing reliance on cloud processing and the continuous transmission of raw sensor data [13,15]. Although the present study did not deploy the evaluated pipelines on embedded or wearable hardware, the results provide an initial comparison of their relative computational demands under a standardized desktop-computing environment.

Within the evaluated conditions, computationally simpler pipelines frequently achieved a predictive performance comparable to that of more complex preprocessing configurations while requiring lower training or inference times. This relative efficiency may be relevant when selecting candidate pipelines for subsequent evaluation on resource-constrained devices. However, desktop-based execution times alone are insufficient to establish suitability for Edge Computing, TinyML, or real-time deployment.

This consideration is particularly important for wearable sensors, which generally operate under constraints related to processing capacity, available memory, battery life, and energy consumption. In such environments, modest improvements in predictive performance may not justify substantial increases in computational complexity. Previous studies have demonstrated the feasibility of deploying behavior-classification models on low-cost embedded devices [57], but such feasibility depends on the characteristics of the hardware, model architecture, preprocessing operations, and implementation framework.

Reducing pipeline complexity may contribute to lower computational and communication requirements and could potentially improve battery life and operational autonomy. Nevertheless, these effects were not directly measured in the present study. Therefore, the computational results should be interpreted as a relative comparison among the evaluated preprocessing pipelines rather than as direct evidence of embedded deployment feasibility.

Previous studies have emphasized that selecting preprocessing strategies for embedded applications should balance predictive performance with computational cost, memory requirements, and energy efficiency according to the characteristics of the target hardware [58]. Accordingly, the computational results obtained in the present study should be interpreted as an initial comparative assessment rather than as validated evidence of embedded deployment feasibility.

Overall, pipelines combining no filtering or computationally simple filtering approaches with Z-score standardization may represent promising candidates for future embedded evaluation because they showed a favorable balance between predictive performance and relative computational cost under the adopted experimental conditions. Further studies should validate these pipelines on actual edge platforms and assess end-to-end latency, preprocessing time, memory consumption, model size, throughput, and energy requirements before conclusions regarding TinyML or real-time field deployment can be established.

### 4.6. Guidelines for Developing Behavior-Classification Pipelines Using Inertial Sensors

The results obtained in this study demonstrate that the definition of pipelines for behavior classification using inertial sensors should consider the interaction among preprocessing strategies, data representation, learning algorithm, and computational requirements of the application. Although steps such as filtering, normalization, feature extraction, and model selection are frequently evaluated individually, limited evidence is still available regarding how these decisions jointly influence the performance and efficiency of classification systems [2,12]. Based on these findings, Table 8 summarizes a set of practical recommendations intended to support the design of behavior-classification pipelines for different application scenarios.

Rather than identifying a universally optimal pipeline, the present findings indicate that preprocessing decisions should be tailored to the characteristics of the monitored signals, the learning algorithm, the chosen data representation, and the computational constraints of the target application. This context-dependent perspective explains why strategies such as omitting filtering or applying Z-score standardization consistently provided favorable trade-offs across several evaluated scenarios, while other decisions depended more strongly on the classification model.

The recommendations summarized in Table 7 are intended as practical guidelines rather than prescriptive rules. Their purpose is to support the development of efficient behavior-classification systems while recognizing that pipeline design should remain flexible according to the requirements of each application. This perspective is particularly relevant for PLF systems based on Edge Computing and TinyML, where predictive performance must be balanced with computational efficiency and implementation feasibility.

### 4.7. Limitations and Future Perspectives

Although this study systematically evaluated the influence of filtering, normalization, and data representation strategies using five independent datasets, different sampling frequencies, and multiple machine learning algorithms, some limitations should be considered when interpreting the results.

A first limitation refers to the post hoc statistical comparisons. Although the Friedman test identified significant global differences in several scenarios, multiple comparisons performed using the Wilcoxon signed-rank test with Bonferroni correction did not identify significant differences between specific pairs of strategies. This behavior is probably related to the reduced number of independent observations used in the comparisons (five folds), decreasing the statistical power of the post hoc test and making the Bonferroni correction more conservative in scenarios involving multiple comparisons [59,60]. Therefore, the results should be interpreted by jointly considering statistical significance, effect sizes, and the consistency of the patterns observed across different datasets, algorithms, and experimental configurations.

Another limitation concerns the performance metric adopted in this study. The weighted F1-score was selected as the primary evaluation metric because the objective was to compare the relative effects of preprocessing strategies while preserving the original class distributions observed in realistic livestock-monitoring datasets. However, because weighted F1-score assigns greater influence to more frequent behavioral classes, it may underrepresent the classification performance of rare behaviors, particularly in highly imbalanced datasets such as Dataset 5. Consequently, the reported results should be interpreted primarily as a comparative assessment of preprocessing strategies rather than as a comprehensive evaluation of the recognition performance of individual behavioral classes. Future studies should complement this comparative analysis using class-wise metrics, Macro F1-score, balanced accuracy, and confusion matrices when the objective is to investigate the detection of infrequent behaviors.

An additional limitation concerns the characteristics of the evaluated datasets and the adopted validation procedures. Unlike Datasets 3–5, Datasets 1 and 2 did not provide individual animal identifiers or recording-session metadata, preventing the application of group-based validation strategies. Consequently, the validation protocols adopted in the original studies were maintained to preserve methodological consistency and comparability with previously published results. In addition, due to its large size, Dataset 4 required a stratified temporal-block subsampling procedure to make the computational experiments feasible. Although this procedure preserved temporal continuity, behavioral diversity, and animal representation, only one stratified subsample was generated. Therefore, future studies should investigate whether the observed preprocessing effects remain consistent across multiple independently generated subsamples. Future studies should also reassess Datasets 1 and 2 using group-based or temporally separated validation schemes whenever such metadata become available.

Another limitation concerns the preprocessing strategies evaluated. Although filters frequently employed in accelerometer signal processing were selected, including Moving Average, Butterworth, and high-pass filters, other potentially relevant approaches, such as adaptive filters, wavelet-based techniques, and machine learning-driven noise removal methods, were not investigated. In addition, filter parameters were standardized using a fixed number of samples (Moving Average) and normalized cutoff frequencies (Butterworth). Consequently, the corresponding temporal durations and cutoff frequencies in Hz differed according to the sampling frequency of each dataset. Although this design reflects common digital signal processing practice, future studies should investigate whether biologically equivalent filtering configurations lead to similar conclusions across datasets acquired at different sampling rates. Likewise, feature selection was based exclusively on Mean Decrease in Impurity (MDI) estimated from Random Forest models. Although this approach is computationally efficient and widely adopted, it may introduce bias in variable importance estimates, and the selected feature subsets are expected to vary across datasets and cross-validation folds according to the characteristics of the training data. Future studies should therefore compare alternative feature importance methods, such as permutation importance or SHAP-based approaches, and investigate the stability of feature selection across different experimental scenarios. These alternative filtering approaches may also present advantages in specific scenarios, especially for noisy or non-stationary temporal signals, due to their greater ability to adapt to the characteristics of the analyzed data [61,62].

In addition, this study focused exclusively on signals obtained from triaxial accelerometers. Although accelerometers represent the most widely used sensors in animal behavior-classification systems [2], integration with gyroscopes, magnetometers, and other sensors may provide complementary information and improve the identification of more complex behaviors [63,64].

Finally, four classification algorithms were evaluated, including three traditional methods (Random Forest, SVM, and KNN) and one deep learning-based architecture (1D-CNN). Although these models represent different learning paradigms and are widely used in applications involving inertial sensors, more recent architectures, such as Transformers, LSTM, Graph Neural Networks, hybrid models, and AutoML-based techniques, were not evaluated in this study [65,66,67].

Therefore, the results should be interpreted considering the scope of the evaluated datasets, preprocessing strategies, and learning algorithms. Nevertheless, the findings also highlight that defining behavior-classification pipelines remains an open challenge, as preprocessing effectiveness depends on the interaction among signal characteristics, data representation, learning algorithms, and deployment environments.

A promising direction is the development of adaptive preprocessing pipelines, in which filtering, normalization, and data representation are automatically adjusted according to signal characteristics, monitored behaviors, and deployment constraints. Likewise, end-to-end learning approaches capable of jointly optimizing feature representation and preprocessing during training may reduce dependence on manually designed pipelines and improve generalization across different monitoring scenarios.

Another important perspective involves the evaluation of new machine learning architectures applied to time series obtained from inertial sensors. Models based on Transformers, LSTM, Graph Neural Networks, hybrid architectures, and AutoML-based approaches may contribute to the development of systems capable of automatically selecting efficient combinations among preprocessing, data representation, and classification algorithms.

From an instrumentation perspective, future research should explore the integration of different sensors, such as accelerometers, gyroscopes, and magnetometers, as well as validate pipelines across different species, production systems, and environmental conditions. This step will be essential to evaluate the generalization capacity of models and bring behavior-classification systems closer to the real-world conditions encountered in precision livestock farming.

Finally, advances in Edge Computing and TinyML create opportunities for developing pipelines specifically optimized for embedded devices, simultaneously considering predictive performance, energy consumption, available memory, latency, and operational autonomy. The integration of smart sensors, compact models, and local processing represents an important step toward transforming machine learning-based behavior classification into practical tools for continuous real-time livestock monitoring.

## 5. Conclusions

This study demonstrated that the effects of preprocessing strategies in accelerometer-based behavior-classification systems are not universal but depend on the interaction among machine learning algorithm, data representation, and signal characteristics. The application of filters did not necessarily result in performance improvements, with the absence of filtering achieving equivalent or superior results in most evaluated scenarios, whereas the high-pass filter was associated with lower performance and higher computational cost.

Among normalization strategies, Z-score standardization showed the most robust behavior, particularly for algorithms sensitive to feature scaling, such as SVM, KNN, and 1D-CNN. Conversely, Random Forest presented high stability regardless of the preprocessing strategy applied. In addition, selected features consistently favored conventional machine learning algorithms, whereas the optimal data representation for the 1D-CNN depended on the adopted preprocessing strategy, with raw signals showing advantages under specific normalization settings and selected features remaining competitive under most filtering conditions.

Considering predictive performance and computational efficiency together, simpler pipelines based on the absence of filtering or smooth filters combined with Z-score standardization represent efficient alternatives for animal monitoring applications using inertial sensors. These findings reinforce that the development of precision livestock farming systems should prioritize pipelines tailored to the interaction among the learning algorithm, data representation, preprocessing strategy, and computational constraints of the target application, rather than relying on standardized preprocessing workflows.

## Figures and Tables

**Table 1:** Characteristics of the accelerometer datasets used to evaluate filtering and normalization strategies.

Dataset	Sampling Frequency	Sensor Model	Species	Sensor Location	Number of Animals	Number of Samples	Behavioral Classes (Samples)	Source
Dataset 1	1 Hz	ADXL345	Sheep	Halter (jaw region)	18	33,769	Feeding (11,210), Rumination (11,401), Other behaviors (11,158)	Amorim et al. [19]
Dataset 2	1 Hz	ADXL345	Cattle	Leg-mounted sensor	5	22,110	Feeding (3684), Lying (4392), Standing (2712), Lying down (1962), Standing up (1824), Normal walking (4434), Active walking (3102)	Wang et al. [20] Available at: https://doi.org/10.1371/journal.pone.0203546.s001 (accessed on 12 August 2026)
Dataset 3	10 Hz	ADXL362	Cattle	Nose ring	7	1,252,536	Feeding (359,711), Rumination (294,985), Standing (1827), Lying (3368), Walking (592,645)	Feng et al. [21] Available at: https://www.kaggle.com/datasets/fandaoerji/cow-nose-ring-data-set (accessed on 12 August 2026)
Dataset 4	25 Hz	Axivity AX3	Goats	Horn	59	1,020,896	Displacement (32,388), Grazing (250,000), Rumination (250,000), Resting (250,000), Other (238,508)	Faillot, Troupe and Bonneau [22] Available at: https://doi.org/10.57745/CYF9QW (accessed on 12 August 2026)
Dataset 5	25 Hz	KX122-1037	Cattle	Neck collar	6	1,204,663	Attacking (366), Being mounted (54), Drinking (7201), Escaping (128), Feeding in Stanchion (19,844), Grazing (17,613), Licking (1302), Moving (51,129), Resting in lying position (764), Resting in standing position (182,880), Ruminating in standing position (110,769), Salt licking (43,840), Urinating (621), Other (768,152)	Ito et al. [23]. Available at: https://zenodo.org/records/5399259 (accessed on 12 August 2026)

**Table 2:** Experimental preprocessing scenarios.

Filtering	Normalization
None	None
None	Min-Max
None	Z-score
Moving Average	None
Moving Average	Min-Max
Moving Average	Z-score
Butterworth LPF	None
Butterworth LPF	Min-Max
Butterworth LPF	Z-score
High-pass	None
High-pass	Min-Max
High-pass	Z-score

**Table 3:** Equivalent temporal duration of the Moving Average filter and Butterworth cutoff frequencies according to the sampling frequency of each dataset.

Dataset	Sampling Frequency (Hz)	Moving Average Window	Equivalent Duration (s)	Low-Pass Cutoff (Hz)	High-Pass Cutoff (Hz)
1	1	3 samples	3.00	0.15	0.025
2	1	3 samples	3.00	0.15	0.025
3	10	3 samples	0.30	1.50	0.250
4	25	3 samples	0.12	3.75	0.625
5	25	3 samples	0.12	3.75	0.625

Moving Average duration was calculated from the fixed three-sample window used in all datasets. Butterworth cutoff frequencies correspond to the normalized cutoff frequencies adopted in this study (0.30 for the low-pass filter and 0.05 for the high-pass filter), expressed in Hz according to the sampling frequency of each dataset.

**Table 4:** Summary of the effect of filtering strategies on different machine learning models.

Dataset	Random Forest	SVM	KNN	1D-CNN
Dataset 1	6/6 (W= 0.81)	6/6 (W = 0.89)	6/6 (W = 0.92)	5/6 (W = 0.74)
Dataset 2	6/6 (W = 0.93)	6/6 (W = 0.85)	6/6 (W = 0.90)	6/6 (W = 0.69)
Dataset 3	0/6 (W = 0.26)	3/6 (W = 0.51)	4/6 (W = 0.67)	0/6 (W = 0.23)
Dataset 4	6/6 (W = 0.76)	4/6 (W = 0.65)	4/6 (W = 0.73)	4/6 (W = 0.55)
Dataset 5	3/6 (W = 0.56)	6/6 (W = 0.69)	3/6 (W = 0.57)	3/6 (W = 0.51)

Values represent the number of scenarios with significant differences among filters (Friedman test, *p* < 0.05) relative to the total number of scenarios evaluated for each Dataset × Model combination. Values in parentheses correspond to the average effect size (Kendall’s W).

**Table 5:** Summary of the effect of normalization strategies on different machine learning models.

Dataset	Random Forest	SVM	KNN	1D-CNN
Dataset 1	0/8 (W = 0.15)	8/8 (W = 0.87)	3/8 (W = 0.47)	5/8 (W = 0.60)
Dataset 2	1/8 (W = 0.27)	6/8 (W = 0.69)	1/8 (W = 0.35)	6/8 (W = 0.70)
Dataset 3	0/8 (W = 0.14)	5/8 (W = 0.64)	1/8 (W = 0.33)	3/8 (W = 0.53)
Dataset 4	0/8 (W = 0.14)	8/8 (W = 0.82)	5/8 (W = 0.61)	5/8 (W = 0.70)
Dataset 5	1/8 (W = 0.19)	5/8 (W = 0.78)	2/8 (W = 0.35)	5/8 (W = 0.60)

Values represent the number of scenarios with significant differences among normalization strategies (Friedman test, *p* < 0.05) relative to the total number of scenarios evaluated for each Dataset × Model combination. Values in parentheses correspond to the average effect size (Kendall’s W).

**Table 6:** Best combinations between filtering and normalization strategies.

Model	Best-Performing Combination	F1-Score	Worst Combination	F1-Score	Improvement (%)
RF	No filter + any normalization	0.773	High-pass	0.741	4.3
SVM	Moving Average + Z-score	0.720	High-pass + no normalization	0.584	23.3
KNN	Moving Average + Z-score	0.709	High-pass + no normalization	0.594	19.4
CNN	No filter + Z-score	0.717	High-pass + Min-Max	0.567	26.5

**Table 7:** Summary of the influence of preprocessing strategies on computational efficiency.

Metric	Filtering	Normalization	Greater Influence
Training time	65/120 (0.53)	49/160 (0.43)	Filtering
Inference time	54/120 (0.53)	53/160 (0.45)	No clear difference

Values represent the number of scenarios with significant differences (Friedman test, *p* < 0.05) relative to the total number of evaluated scenarios. Values in parentheses correspond to the average effect size (Kendall’s W).

**Table 8:** Guidelines for developing behavior-classification pipelines using inertial sensors.

Application Objective	Recommended Strategy	Justification
Maximize overall performance	Z-score normalization with no filtering or low-pass smoothing	Consistently among the highest-performing combinations while maintaining robustness and low computational cost
Embedded systems	No filter + Z-score	Lower processing requirements while maintaining high accuracy
Minimize computational cost	No filter or Moving Average	Lower training and inference times
Random Forest	Normalization optional	Low sensitivity to feature scaling
SVM	Z-score recommended	High dependence on feature scale
KNN	Z-score recommended	Distances become more representative
1D-CNN	Select the data representation according to the preprocessing strategy	Raw signals performed better under some normalization strategies, whereas selected features remained competitive under most filtering conditions
Conventional algorithms	Use selected features	Greater overall robustness across preprocessing strategies
Use with caution	High-pass filter	Consistently associated with lower performance in the evaluated scenarios

## Data Availability

The datasets analyzed in this study are publicly available. The sources and access information for each dataset are provided in Table 1. No new experimental data were generated in this study.

## Supplementary Materials

The following supporting information can be downloaded at: https://www.mdpi.com/article/10.3390/s26175353/s1, Table S1: Extracted features and mathematical formulation of accelerometer-derived metrics; Table S2: Complete Macro F1-score and balanced accuracy results for all combinations of datasets, preprocessing strategies, data representations, and machine learning models; Table S3: Complete performance results obtained for all combinations of datasets, preprocessing strategies, data representations, and machine learning models; Table S4: Complete statistical analysis results, including Friedman tests, Wilcoxon post hoc comparisons, and Kendall’s W effect sizes; Table S5: Complete computational efficiency results, including preprocessing time, training time, and inference time for all evaluated pipelines.

## Abbreviations

1D-CNN	One-Dimensional Convolutional Neural Network
FFT	Fast Fourier Transform
GPU	Graphics Processing Unit
Kendall’s W	Kendall’s Coefficient of Concordance
KNN	k-Nearest Neighbors
LPF	Low-Pass Filter
MDI	Mean Decrease in Impurity
ML	Machine Learning
ODBA	Overall Dynamic Body Acceleration
PLF	Precision Livestock Farming
RBF	Radial Basis Function
RF	Random Forest
RMS	Root Mean Square
SMA	Signal Magnitude Area
SVM	Support Vector Machine
TinyML	Tiny Machine Learning
VeDBA	Vectorial Dynamic Body Acceleration
VM	Signal Vector Magnitude
