# Supplementary material for: Preprocessing Strategies for Animal Behavior Classification Using Inertial Sensors: Effects of Filtering, Normalization, and Data Representation
Source: Sensors (Basel). 2026 Aug 24;26(17):5353. doi: 10.3390/s26175353 (PMC13568227; doi:10.3390/s26175353)
Supplement: Supplementary file 1 [file sensors-26-05353-s001.zip › Supplementary_Table_S1.pdf]

Supplementary Table S1. Extracted features used in this study and mathematical formulation of accelerometer-derived metrics.

Feature group	Feature	Description	Domain
Statistical (per axis X, Y, Z)	Mean	Average acceleration value within the window	Time
	Standard deviation	Dispersion of acceleration values around the mean	Time
	Minimum	Smallest acceleration value in the window	Time
	Maximum	Largest acceleration value in the window	Time
	Range	Difference between maximum and minimum values	Time
	Variance	Variability of the acceleration signal	Time
	Median	Central value of the acceleration distribution	Time
	RMS	Root Mean Square of the acceleration signal	Time
	Skewness	Measure of asymmetry of the signal distribution	Time
	Kurtosis	Measure of the peakedness of the signal distribution	Time
Vector magnitude	Signal Vector Magnitude (SVM)	Combined magnitude of the three acceleration axes	Time
Statistical (SVM)	Mean	Average magnitude of acceleration	Time
	Standard deviation	Dispersion of magnitude values	Time
	Minimum	Minimum magnitude value	Time
	Maximum	Maximum magnitude value	Time
	Range	Difference between max and min magnitude	Time
	Variance	Variability of magnitude values	Time
	Median	Median magnitude value	Time
	RMS	Root Mean Square of magnitude signal	Time
	Skewness	Asymmetry of magnitude distribution	Time
	Kurtosis	Peakedness of magnitude distribution	Time
Movement intensity	SMA	Signal Magnitude Area representing overall activity level	Time
	ODBA	Overall Dynamic Body Acceleration representing dynamic movement	Time

	VeDBA	Vectorial Dynamic Body Acceleration measuring movement intensity	Time
Spectral features	Spectral energy	Total energy of the signal in the frequency domain	Frequency
	Spectral entropy	Measure of signal complexity in the frequency domain	Frequency
	Dominant frequency	Frequency with the highest power in the spectrum	Frequency

**Feature**	**Equation**
Signal Vector Magnitude (SVM)	$SVM = \sqrt{x_i^2 + y_i^2 + z_i^2}$
Signal Magnitude Area (SMA)	$SMA = \frac{1}{N}\sum_{i=1}^{N}(\lvert x_i\rvert + \lvert y_i\rvert + \lvert z_i\rvert)$
Overall Dynamic Body Acceleration (ODBA)	$ODBA = \sum_{i=1}^{N}(\lvert x_i - \bar{x}\rvert + \lvert y_i - \bar{y}\rvert + \lvert z_i - \bar{z}\rvert)$
Vectorial Dynamic Body Acceleration (VeDBA)	$VeDBA = \sqrt{(x_i - \bar{x})^2 + (y_i - \bar{y})^2 + (z_i - \bar{z})^2}$
